# The quest for optimal femoral torsion angle measurements: a comparative advanced 3D study defining the femoral neck axis

**DOI:** 10.1186/s40634-023-00679-9

**Published:** 2023-12-18

**Authors:** Bert Van fraeyenhove, Jeroen C. F. Verhaegen, Jonas Grammens, Gino Mestach, Emmanuel Audenaert, Annemieke Van Haver, Peter Verdonk

**Affiliations:** 1 Universitair Ziekenhuis Antwerpen, Antwerp, Belgium; 2 University of Antwerp, Antwerp, Belgium; 3 Universitair Ziekenhuis Gent, Ghent, Belgium; 4 AZ Monica, Antwerp, Belgium; 5 Orthoca, Kielsevest 14, Antwerp, 2018 Belgium; 6 MoRe Institute, 2100 Antwerp, Belgium; 7 Department of Orthopaedic Surgery, Antwerp University, 2000 Antwerp, Belgium

**Keywords:** Hip, Femoral torsion, 3D, CT, Femoral neck axis

## Abstract

**Purpose:**

There is high variability in femoral torsion, measured on two-dimensional (2D) computed tomography (CT) scans. The aim of this study was to find a reliable three-dimensional (3D) femoral torsion measurement method, assess the influence of CAM deformity on femoral torsion measurement, and to promote awareness for the used measurement method.

**Methods:**

3D models of 102 dry femur specimens were divided into a CAM and non-CAM group. Femoral torsion was measured by one 2D-CT method described by Murphy et al. (method 0) and five 3D methods. The 3D methods differed in strategies to define the femoral neck axis. Method 1 is based on an elliptical least-square fit at the middle of the femoral neck. Methods 2 and 3 defined the centre of mass of the entire femoral neck and of the most cylindrical part, respectively. Methods 4 and 5 were based on the intersection of the femoral neck with a 25% and 40% enlarged best fit sphere of the femoral head.

**Results:**

3D methods resulted in higher femoral torsion measures than the 2D method; the mean torsion for method 0 was 8.12° ± 7.30°, compared to 9.93° ± 8.24° (*p* < 0.001), 13.21° ± 8.60° (*p* < 0.001), 8.21° ± 7.64° (*p* = 1.00), 9.53° ± 7.87° (*p* < 0.001) and 10.46° ± 7.83° (*p* < 0.001) for methods 1 to 5 respectively. In the presence of a CAM, torsion measured with method 4 is consistently smaller than measured with method 5.

**Conclusion:**

2D measurement might underestimate true femoral torsion and there is a difference up to 5°. There is a tendency for a higher mean torsion in hips with a CAM deformity. Methods 4 and 5 are the most robust techniques. However, method 4 might underestimate femoral torsion if a CAM deformity is present. Since method 5 is independent of a CAM deformity, it is the preferred technique to define expected values of torsion.

## Background

The femoral torsion refers to the twist or torsion between the proximal and distal femur and is typically measured in the transverse plane as the angle between the femur neck axis (FNA) and the posterior condyle line (PCL) of the distal femur [14]. Variations in femoral torsion play an important role in the biomechanics of the hip and knee joint. Increased torsion of the femoral neck is often observed in combination with hip dysplasia and associated with instability [23] and it has been recognised as a cause for posterior ischiofemoral impingement [34]. Femoral retrotorsion in combination with acetabular retroversion can cause femoroacetabular impingement (FAI) [11, 37]. At the level of the knee, increased femoral torsion leads to increased patellofemoral pressures causing anterior knee pain [22, 42], patellofemoral instability [7, 8] and has been associated with anterior cruciate ligament injury [1, 16].

Reliable assessment of the femoral torsion is not only important for diagnostics and understanding of pathological processes, but also for planning of hip and knee surgery [9, 10, 38, 43]. Femoral torsion assessments are incorporated in three-dimensional (3D) planning software for knee and hip arthroplasty, in which expected values are targeted to obtain optimal femoral component orientation and rotational alignment [20, 28].

A wide range of expexted reference values has been reported in literature on femoral torsion, ranging from 10° to 25° [23, 37]. These expected values are not used adequately and this is likely the consequence of highly variable morphology of the proximal femur, and thus FNA, and an inconsistent use of a large number of measuring techniques. In addition, the complex 3D structure of the femoral neck poses difficulties measuring the femur neck axis consistently. The PCL is a consistent axis to define the distal femur and the measured torsion mainly depends on the chosen FNA [15, 33]. Defining the FNA in a standardized way is challenging, particularly when common local morphological deviations like a CAM morphology are present. A CAM deformity is an aspherical anterior prominence at the femoral head-neck junction which may substantially affect the assessment of the femoral neck axis. The prevalence of a CAM morphology has been estimated at 34% of males and 20% of female subjects [13, 27]. The prevalence of such abnormalities has been reported to be as high as 17–35%, and multiple studies have shown a significant relationship between radiological parameters specific to FAI and the development of OA of the hip [17, 19].

In clinical settings, femoral torsion is conventionally measured on a computed tomography (CT) scan in which the femoral neck runs obliquely over multiple transverse slices. Therefore, the femoral FNA can theoretically not be determined on a single transverse image. To overcome this problem, Murphy suggested to define the FNA on a selection of two transverse 2D slices.

Recently, new 3D imaging modalities have gained popularity over the 2D approach and have been implemented in 3D planning software. However, lack of consistency, clarity, and awareness of differences in measurement techniques still hampers clinical application [15, 33].

The aim of this study was (1) to compare five different 3D FNA, and therefore femoral torsion, measurement methods against the gold standard 2D-CT technique as described by Murphy [30] in a group of CT-based 3D bone models of 102 dry femur specimens, (2) to assess how femoral torsion measurement is influenced by the presence of a CAM lesion and (3) to create awareness for the used measurement technique.

## Methods

### Study design

The dataset that was used comprises 102 dry femur specimens obtained from the osteological library of the Vrije Universiteit Brussel. Femurs showed no macroscopic evidence of dysplasia or major arthritic changes. This dataset has previously been used in a study on a 3D detection method of CAM deformities by Audeneart et al. [2].

The included 3D bone models were based on 64-slice Computed Tomography (CT) scans (Light-Speed VCT, GE Healthcare, Milwaukee, WI) with a 0.63 mm slice increment and a pitch of 0.97:1 at 120 kV. Pixel size was 0.79 × 0.79 mm. A density-based automated segmentation was performed using the Mimics® software package (Materialise NV, Heverlee, Belgium) to obtain 3D images [2].

A validated fully automated computational analysis of the femurs was conducted to divide them into a CAM and non-CAM group. As a result 32/102 (31.37%) femurs were classified with a CAM deformity and 70/102 (68.63%) without a CAM deformity [2].

### Measurement techniques

Femoral torsion was defined as the angle between the FNA and the PCL projected on a plane perpendicular to the femoral shaft axis [18].

One author (J.V.) measured femoral torsion using Murphy’s method, the standard 2D-CT method. Following a literature search on computational measurement techniques and surgical navigation, five different 3D methods to measure femoral torsion, were selected [2, 3, 5, 6, 41]. One existing computational method (method 1) [2] and two methods based on surgical navigation in robot-assisted total hip arthroplasty (method 2–3) [6], in which the surgeon defines the femoral head and neck surface by means of a spatially tracked pointer device, were used. In addition, two newly developed methods (method 4–5) were applied.

For all five 3D methods, the PCL was defined as the line connecting the two most posterior points on the posterior condyles. The FNA was defined as the line connecting the centroids of the femur head and the femur neck. The centre of the femoral head was defined as the centre of the best fitting sphere in the femoral head and was the same for all 3D methods. The definition of the centre of the femur neck was different in each of the five 3D methods and is described for each method separately. While the definition of the femoral neck centre in method 1–3 is highly standardized, these methods depend on the availability of the specific computational method and are therefore more difficult to implement by different user groups. To make a standardized 3D approach more accessible for a broader user group, alternative methods (method 4–5) were explored. These methods can be applied both computationally and manually in medical 3D software packages.

#### Method 0: conventional 2D measurement

FNA following Murphy’s method is defined on a selection of two transverse 2D slices of the CT scan [30]. The first transverse slice defines the location of the centre of the femoral head. This centre is then connected to the centre of the base of the femoral neck, identified on a second transverse slice directly superior to the lesser trochanter, resulting in the FNA [23, 32]. PCL was defined as the tangent to the posterior condyles on a single transverse image in which the condyles had their maximum expansion from anterior to posterior [30].

#### Method 1

The first 3D measurement method was adopted from Audenaert et al. [2], who described the femoral neck as an ellipse on a cross section. An elliptical least-square fitting approach was applied to the mid-neck area and the femoral neck centre was defined as the cross-sectional centre of the femoral neck.

#### Method 2

**Fig. 1:**
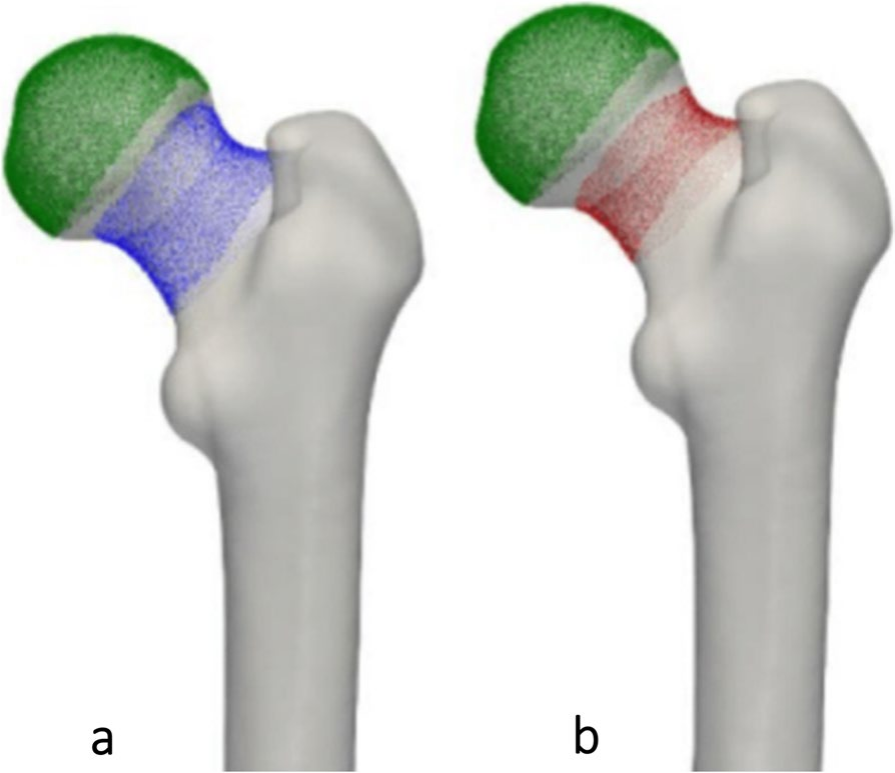
Methods 2 and 3 to assess femoral neck using 3D technology. **a** Method 2: Centre of mass of the complete femoral neck surface. **b** Method 3: Centre of mass of the most cylindrical part of the femoral neck surface

This is a simulation of surgical navigation in robot-assisted total hip arthroplasty, in which the surgeon defines the neck surface by means of spatially tracked pointer device [6]. In method 2, the centre of mass of the complete femoral neck surface was calculated (Fig. 1).

#### Method 3

Method 3 is also a simulation of surgical navigation in robot-assisted total hip arthroplasty [6]. In this method, the most cylindrical part of the femoral neck surface was selected and the centre of mass of these surface points defined the femur neck centre (Fig. 1).

#### Method 4

**Fig. 2:**
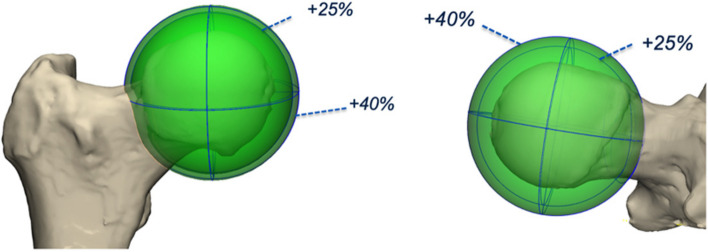
Methods 4 and 5 to assess femoral neck using 3D technology. A best fit sphere of the femoral head was drawn. Next, a second sphere was generated by increasing the radius with 25% (method 4) and 40% (method 5)

Method 4 is a novel technique in which a best fit sphere on the femoral head was enlarged with 25%, resulting in an intersection of this enlarged sphere and the femoral neck. The best fit arc of this intersection was created and the centre of this arc was identified as the centre of the femur neck (Fig. 2).

#### Method 5

Method 5 is a variation of method 2, applying a 40% increase of the radius of the best fit sphere (Fig. 2).

### Statistics

All measurements are reported as mean ± standard deviation (SD). Parametric tests were used after exploratory data analysis using Q-Q plots and Kolmogorov–Smirnov tests (*p* = 0.200) showing a normal distribution for both the CAM group and the non-CAM group.

To evaluate the difference and the correlation between the femoral torsion measurement methods repeated measures analysis of variance (ANOVA) with additional post hoc Bonferroni and Pearson correlation analysis were applied. Correlations were graded as poor (*R* ≤ 0.3), fair (*R* = 0.31–0.5), moderate (*R* = 0.51–0.6), moderately strong (*R* = 0.61–0.8), or very strong (*R* = 0.81–1) [12].

To detect differences in femoral torsion between the CAM group and non-CAM group an independent-samples t-test was performed.

The data were analysed using IBM SPSS Statistics, Version 27.0. A *p*-value of < 0.05 was considered significant.

## Results

**Fig. 3:**
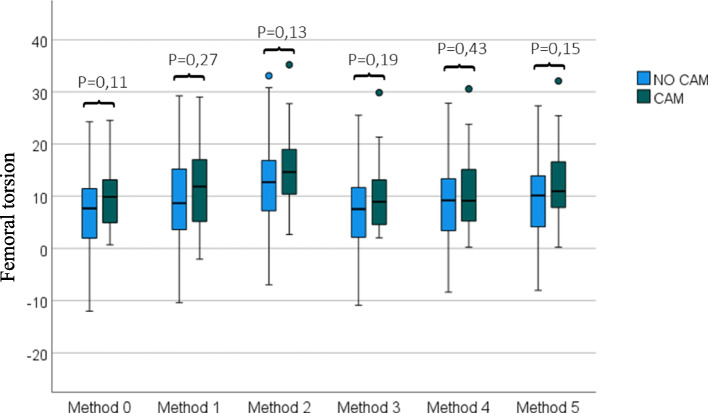
Femoral torsion (°), measured with one 2D method (Method 0) and 5 different 3D methods in the NO CAM and CAM group. Independent-samples t-test showed no statistically significant difference between non-CAM and CAM group

The lowest mean femoral torsion was found for methods 0 and 3 with a mean torsion of 8.12° ± 7.30° (range: -12.02°–24.54°) and 8.21° ± 7.64° (range: -10.89°–29.86°) respectively (Fig. 3, Table 1). The highest values were found for method 2 with a mean value of 13.21° ± 8.60° (range: -6.94°–35.21°). The greatest difference in the mean torsion was found between method 0 and 2 with a mean difference of 5.10° ± 3.07° (*p* < 0.001), and between method 2 and 3 with a mean difference of 5.00° ± 2.44° (*p* < 0.001).

**Table 1:** Femoral torsion measurements for the 6 methods (mean ± SD) with *p*-values for repeated measures ANOVA with a Greenhouse–Geisser correction

Measurement method	No CAM (*n* = 70)	CAM (*n* = 32)	Total (*n* = 102)
Method 0	7.32° ± 7.74°	9.85° ± 5.96°	8.12° ± 7.30°
Method 1	9.32° ± 8.37°	11.26° ± 7.93°	9.93° ± 8.24°
Method 2	12.34° ± 9.18°	15.13° ± 6.95°	13.21° ± 8.60°
Method 3	7.54° ± 8.13°	9.69° ± 6.30°	8.21° ± 7.64°
Method 4	9.11° ± 8.26°	10.45° ± 6.98°	9.53° ± 7.87°
Method 5	9.70° ± 8.16°	12.12° ± 6.87°	10.46° ± 7.83°
P-value	< 0.001^*^	< 0.001^*^	< 0.001^*^

^*^ P < 0.05

Overall, there was a significant difference in femoral torsion between measurement methods as determined by repeated measures ANOVA with a Greenhouse–Geisser correction (*p* < 0.001). Bonferroni post hoc test revealed that all comparisons between measurement methods were statistically significant (*p* < 0.001), except for comparisons between method 0 and 3 (*p* = 1), method 1 and 4 (*p* = 1), and between method 1 and 5 (*p* = 1). Differences between the measurement methods in the non-CAM and CAM cohorts separately were statistically significant (*p* < 0.001). Analysis with Bonferroni post hoc test in the non-CAM cohort, demonstrated statistically significant differences between all measurement methods (with *p* ≤ 0.003), except for comparisons between method 0 and 3 (*p* = 1), between method 1 and 4 (*p* = 1), and between method 1 and 5 (*p* = 1). In the CAM group, a significant difference (*p* < 0.001) in femoral torsion was observed between method 2 and methods 0, 1, 3, and 4. A similar statistically significant difference (*p* < 0.001) was observed between method 5 and methods 0, 2, 3, and 4 (Table 1).

**Fig. 4:**
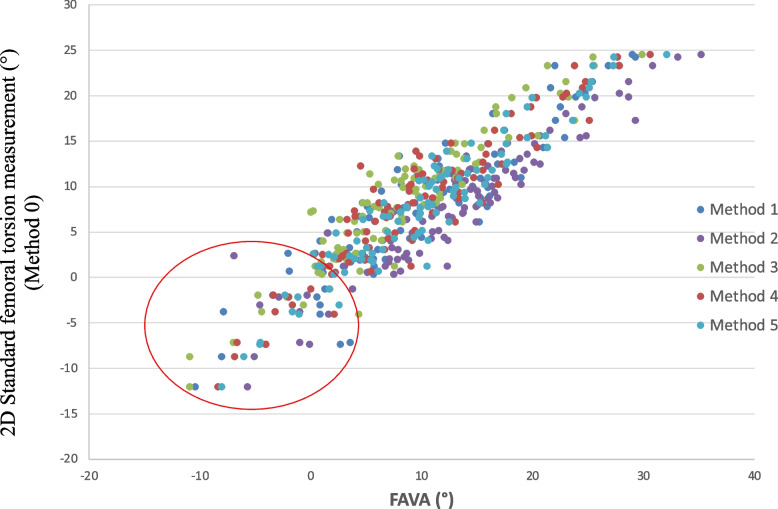
Correlation between methods. Scatter plot demonstrates strong correlation between methods. *R* = 0.90 for method 1, *R* = 0.94 for method 2, *R* = 0.94 for method 3, *R* = 0.93 for method 4, and *R* = 0.95 for method 5, *p* < 0.001. Strongest correlations were seen if torsion > 7.5° as measured with method 0. The red circle illustrates a decreased correlation at lower torsion

In general, very strong correlations between method 0 and the different 3D measurement methods (method 1–5) were observed (Fig. 4). When the torsion was less than 7.5°, correlations between the method 0 and all 3D measurement techniques became slightly weaker but remained strong (*R* = 0.67 for method 1, *R* = 0.78 for method 2, *R* = 0.82 for method 3, *R* = 0.85 for method 4, and *R* = 0.86 for method 5, *p* < 0.001). Correlations between method 0 and the different 3D measurement methods (method 1–5), overall revealed a slightly stronger correlation in the non-CAM cohort (*R* = 0.91 for method 1, *R* = 0.94 for method 2, *R* = 0.94 for method 3, *R* = 0.95 for method 4, and *R* = 0.95 for method 5, *p* < 0.001) compared to the CAM cohort (*R* = 0.89 for method 1, *R* = 0.95 for method 2, *R* = 0.92 for method 3, *R* = 0.90 for method 4, and *R* = 0.94 for method 5, *p* < 0.001).

Femurs with a CAM deformity had on average consistently higher femoral torsion angles, independent of the used measurement technique, but this difference was not statistically significant and there was an important overlap (*p*-values ranging from 0.11 to 0.43) (Fig. 3).

## Discussion

This study indicates that differences in femoral torsion values depend more on the used measurement technique than on the specific patient or patient group. The evaluated 2D measurement technique deviated significantly from four out of the five 3D measurement techniques (methods 1, 2, 4 and 5), indicating that the 2D measurement technique of femoral torsion can be questioned.

Based on these observations, clinicians need to be aware that expected values or threshold values always need to be interpreted relative to the measurement technique used.

Many different 2D measurement techniques exist, but the lack of consistency partly explains a high variability [15, 33]. Murphy’s method uses two transverse slices to determine the axis of the femoral neck and previous research has shown excellent intra- and interobserver correlation for this 2D measurement method [21]. However, a major shortcoming of 2D-CT scans remains the dependency of the measured torsion on the position of the femur during scanning and mainly on the CT segmentation. The measured torsion is significantly reduced when the hip is flexed, which makes it important referencing the CT scan correctly to avoid misleading results [29, 35]. Three-dimensional techniques are independent of femur positioning [25], as they are not limited by the need to define axes on the CT slices, they offer standardization possibilities and can help reducing manual errors in assessing the complex 3D structure of the femoral neck. Therefore, 3D measurement techniques offer a higher accuracy and precision to define the femoral torsion.

In this study, we used 5 different 3D measurement methods to define the femoral neck axis and calculate the femoral torsion, and compared these to a conventional 2D measurement technique as described by Murphy [30]. All 3D measurement techniques defined the FNA as the line between the centre point of the best fit sphere on the femoral head, and a second point in the neck area, which was different for all measurement methods. It is important to note that only one 2D measurement technique was evaluated in this study. As demonstrated by Kaiser et al. [15] the measured femoral torsion depends on the used technique. Therefore, by using only Murphy’s method, correlations between measurement techniques are expressed, rather than average values.

According to Tönnis and Heinecke [37], expected femoral torsion is considered to range between 10° and 25°. These expected values were reported in their review article in 1999, which was based on various studies on patient populations with pathological femoral and acetabular anteversion in relation to pain, hip rotation and osteoarthritis. In these studies, femoral torsion was measured using transverse slices of CT scans. Though more advanced 3D measurement methods have been described in the last decades, these historical expected ranges based on 2D measurements on pathological populations remain to be used [23, 40]. Correlation between 2 and 3D measurement techniques is suboptimal. The femoral torsion showed a trend to a lower expected value as measured with 2D technique (method 0 compared to 3D measurement methods 1, 2, 4 and 5), which questions the value of this 2D technique as golden standard. An alteration in expected torsion is seen between the different 3D techniques. The differences in torsion measured with the various measurement methods, as illustrated in Table 1, are up to 5° and this can be of clinical importance in surgical decision making in femoral derotational osteotomy. The large standard deviations obtained in this study, are also observed in other studies on femoral anteversion [15, 39].

The strongest correlation was observed between method 0 and the 3D measurement methods when femoral torsion was more than 7.5°. When torsion dropped below 7.5°, and especially if less than 0°, the correlation between methods decreased. This requires special attention to accurately diagnose femoral retrotorsion. Although method 2 and 3 are both considered as surgical navigation methods [6], there was a significant difference for torsion measurement between methods 2 and 3 (*p* < 0.001). This difference can be clarified by the use of different neck surface points; in method 2, the centre of mass of the complete femoral neck surface was calculated, whilst in method 3, the centre of mass was obtained from the most cylindrical part of the femoral neck surface. If the less cylindrical part of the neck does not align with the most cylindrical part, this will obviously result in a different definition of the FNA. Furthermore, methods 2 and 3 require a specific computational method and therefore they are more difficult to implement by different user groups. They are less robust compared to method 4 and 5 in which the best fit sphere in the femoral head is enlarged.

We found no significant differences in femoral torsion values comparing femurs with and without a CAM deformity, although there was a tendency for higher torsion values in the CAM group for all different measurement techniques. High femoral torsion has been reported as a possible factor predisposing to the development of a CAM due to altered stress on the femoral physis [31].

**Fig. 5:**
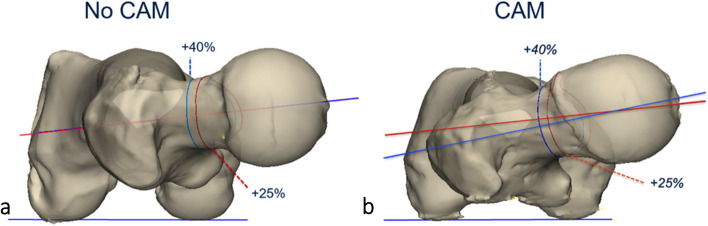
Two 3D reconstructed images demonstrating the difference in torsion between method 4 and 5 in a hip without a CAM and CAM hip. **a** No CAM deformity. The measured torsion is approximately the same for method 4 (red) and 5 (blue). **b** Underestimation of torsion when measured with method 4 as compared with method 5. In method 4, the intersection of the femoral neck is at the level of the CAM, which anteriorizes the centre of the femoral neck and therefore decreases the torsion

Within the CAM group it was observed that out of the two most robust 3D methods (4 and 5), method 5 was the superior one. In method 4 the intersection of the sphere and the femur neck was in some cases located at the level of the CAM, anteriorizing the centre of the femoral neck and risking underestimation of the torsion (Fig. 5). Consequently, method 4 might result in unreliable and misleading results if a CAM deformity is present. In method 5 the intersection of the sphere was for all cases beyond the level of the CAM instead of at the level of the CAM. In this study, expected femoral torsion as measured with method 5 is considered 10° ± 8° for hips without a CAM deformity and 12° ± 7° for hips with a CAM deformity.

We hope the data from this study could help to improve the accuracy of surgical navigation systems for total hip arthroplasty (THA) or in the diagnostic work-up of patients with instability after THA, as femoral torsion affects the risk of dislocation and range of motion [38]. Correct assessment of femoral torsion is also important when considering a femoral derotational osteotomy, for example in young adults with hip pain due to excessive femoral antetorsion or retrotorsion [4, 26], or in patients with malrotation after a diaphyseal femur fracture [24, 36].

Limitations of this study include the use of postmortem femurs of unknown age and gender. Although it is unknown if they had any hip or knee symptoms, none of the selected specimen had macroscopic evidence of dysplasia or major arthritic changes (ie. manifest osteophytes) [2]. Measurements of the FAVA using Murphy’s method were performed by one person, which could be another limitation of the study. Seventy subjects out of 102 (69%) had no CAM deformity and this number might be too low to make assumptions about “expected” femoral version. CT scans only imaged the femur and consequently there is no information about pelvic morphology, which is an important parameter to assess in the young active patient with hip pain [23].

Future research may focus on the interaction between morphological hip parameters, such as femoral head radius, femoral neck length, coxa vara/valga and should take pelvic morphology into account. In addition, it is recommended to perform femoral torsion measurements using method 5 in a larger and asymptomatic cohort of volunteers. In a next stage, focusing on the femoral torsion in dysplastic hips, might be of special interest when planning corrective osteotomies in children.

## Conclusion

Femoral torsion has an important role in hip and knee biomechanics and accurate measurement is crucial in the diagnostic workup and for surgical planning. Conventional 2D measurement techniques are associated with important limitations leading to a high variability of measured values. 3D measurement techniques are independent of patient positioning during scanning and this study shows that the 2D measurement of femoral torsion tends to result in a lower expected value as compared to 3D methods. This means that the correlation between 2 and 3D methods is suboptimal. There is a clear tendency for a higher mean femoral torsion in the CAM group. Methods 4 and 5 are the most robust techniques. However, method 4 might result in misleading values if a CAM deformity is present. Since method 5 is independent of a CAM deformity, it is the preferred technique to define expected values of femoral torsion. Further research in a larger population is necessary to confirm these findings.

## Data Availability

Not applicable.
